# Immunological profile of mice immunized with a polyvalent virosome-based influenza vaccine

**DOI:** 10.1186/s12985-023-02158-0

**Published:** 2023-08-21

**Authors:** Francisco Noé Fonseca, Vanessa Haach, Franciana Volpato Bellaver, Gabrielly Bombassaro, Danielle Gava, Luciano Paulino da Silva, Lana Flavia Baron, Mayara Simonelly, Wanessa Araújo Carvalho, Rejane Schaefer, Ana Paula Bastos

**Affiliations:** 1 Embrapa Suínos e Aves, Concórdia, SC Brazil; 2 Universidade Federal do Rio Grande do Sul, Porto Alegre, RS Brazil; 3 Instituto Federal Catarinense – Campus Concórdia, Concórdia, SC Brazil; 4 Embrapa Recursos Genéticos, Brasília, DF Brazil; 5 Universidade de Brasília, Brasília, DF Brazil; 6 Embrapa Gado de Leite, Juiz de Fora-MG, Juiz de Fora, MG Brazil

**Keywords:** Vaccine, Influenza a virus, Vaccination, Seroprotection, Nanovaccine

## Abstract

**Background:**

Influenza A virus (IAV) causes respiratory disease in pigs and is a major concern for public health. Vaccination of pigs is the most successful measure to mitigate the impact of the disease in the herds. Influenza-based virosome is an effective immunomodulating carrier that replicates the natural antigen presentation pathway and has tolerability profile due to their purity and biocompatibility.

**Methods:**

This study aimed to develop a polyvalent virosome influenza vaccine containing the hemagglutinin and neuraminidase proteins derived from the swine IAVs (swIAVs) H1N1, H1N2 and H3N2 subtypes, and to investigate its effectiveness in mice as a potential vaccine for swine. Mice were immunized with two vaccine doses (1 and 15 days), intramuscularly and intranasally. At 21 days and eight months later after the second vaccine dose, mice were euthanized. The humoral and cellular immune responses in mice vaccinated intranasally or intramuscularly with a polyvalent influenza virosomal vaccine were investigated.

**Results:**

Only intramuscular vaccination induced high hemagglutination inhibition (HI) titers. Seroconversion and seroprotection (> 4-fold rise in HI antibody titers, reaching a titer of ≥ 1:40) were achieved in 80% of mice (intramuscularly vaccinated group) at 21 days after booster immunization. Virus-neutralizing antibody titers against IAV were detected at 8 months after vaccination, indicating long-lasting immunity. Overall, mice immunized with the virosome displayed greater ability for B, effector-T and memory-T cells from the spleen to respond to H1N1, H1N2 and H3N2 antigens.

**Conclusions:**

All findings showed an efficient immune response against IAVs in mice vaccinated with a polyvalent virosome-based influenza vaccine.

**Supplementary Information:**

The online version contains supplementary material available at 10.1186/s12985-023-02158-0.

## Background

The 2009 H1N1 pandemic influenza virus (H1N1pdm09) strongly illustrates the potential of influenza A viruses (IAVs) to cause morbidity and mortality in the human population on a global scale, as well as the importance of swine in the evolution of zoonotic viruses [1]. Pigs are susceptible to infection with both avian and human IAVs, and thus can serve as a “mixing vessel” for the emergence of novel viruses by reassortment of influenza gene segments [2]. IAV is endemic in pigs worldwide, with multiple genetically and antigenically distinct virus lineages of H1N1, H1N2, and H3N2 subtypes circulating in different geographic regions [3, 4]. Clinically, influenza virus infection causes an acute respiratory disease marked by fever, lethargy, coughing, anorexia, and nasal discharge. The IAV disrupts the normal defense system of the respiratory tract and may lead to secondary bacterial infections [5].

The most effective measure to mitigate and control morbidity and mortality associated with IAV in swine populations is vaccination. The swine influenza vaccination is equally crucial for human health, as it reduces swine-to-human and human-to-swine IAV transmission, decreasing the likelihood of pandemic risks and the emergence of new strains [2, 6]. In Brazil, the currently licensed commercial IAV vaccine for pigs is based on the whole inactivated H1N1pdm09 virus (WIV). The protection achieved by this vaccine is primarily mediated by the induction of antibodies targeting the hemagglutinin (HA) and, to a lesser extent, the neuraminidase (NA) viral glycoproteins [7, 8]. However, for a highly effective vaccine, the vaccine antigens must have a close antigenic match with the circulating IAVs in swine herds [9, 10].

The surveillance of IAV in pigs through genetic and antigenic characterization is extremely important for the selection of vaccine candidates. Recently, a great genetic diversity of IAV has been found in the Brazilian pig population, which may have implications for the design of cross-protective vaccines [11]. In this sense, the IAV vaccines for pigs available in the country might provide limited or absent protection against the currently circulating genetically distinct swine IAVs. Furthermore, IAVs have the ability to evade the host immune response through mechanisms known as antigenic drift and antigenic shift, which require a regular update of the viruses that compose the vaccine to match the circulating viruses [12].

Several studies have been conducted in the last few years aiming to develop broadly protective vaccines that induce both humoral [13, 14] and cellular immune responses [3, 7, 15–17]. In general, these vaccines target antigenically conserved epitopes on the HA [18–20], expressed by a virus-like particle (VLP) [21]. Nevertheless, none of the proposed solutions has produced a practical vaccine that induces broad heterosubtypic protection or achieves the desired sterilizing immunity. Broad protection against IAVs can be achieved with either polyvalent vaccines of mixed subtype-specific immunogens or the use of a good immunogen conserved among circulating IAV subtypes [8]. A polyvalent influenza vaccine could decrease the inherent limitations of influenza vaccines because they are designed to protect against different influenza viruses that circulate in swine herds [17, 22]. Furthermore, to achieve effective immunity through immunization, the target of virus-neutralizing antibodies needs to antigenically match the circulating IAVs, which in pigs consist of isolates from distinct lineages of H1N1, H1N2, and H3N2 subtypes [23, 24].

Virosomes are VLPs produced in vitro from purified envelope components; nevertheless, they lack the genetic material and internal proteins of the native virus. Influenza virosomes combine the technical benefits of a well-regulated composition with the immunological advantages of VLPs [25]. Incorporated within the phospholipid bilayer of the virosomes are the functional IAV envelope glycoproteins, HA and NA. These viral proteins not only offer structural stability and homogeneity to the virosomal formulations, but they also contribute significantly to the immunological features of the virosomes, which distinguish them from other liposomal systems [26]. The fully functional fusion activity of virosomes containing the HA protein permits receptor-mediated uptake and natural intracellular processing of the antigen, thereby triggering both humoral and cellular immune responses [27]. In this study, a trivalent virosomal swIAV vaccine based on a H1N1pdm, H1N2 and H3N2 viruses was constructed using a dialyzable short-chain phospholipid (1,2-Dicaproyl-sn-Glycero-3-Phosphocholine, DCPC) as a solubilizing agent, since this surfactant presents the best performance for viral solubilization; it maintains the envelope proteins functionality, and is completely removed by dialysis during virosome production [28]. In addition, virosomes efficacy was demonstrated in mice by measuring the cellular immune response and the serum antibody response against the IAV vaccine strains.

## Methods

### Viruses

Three IAVs isolated from swine were selected for the preparation of the influenza vaccine: A/swine/Brazil/025 − 15/2015 1 A.3.3.2 (H1N1pdm; NCBI GenBank Accession HA = MH559931 and NA = MH559933; BRMSA 1710), A/swine/Brazil/223-15-1/2015 1B.2.4 (H1N2; NCBI GenBank Accession HA = MH560035 and NA = MH560037; BRMSA 1698) and A/swine/Brazil/028-15-8/2015 (H3N2; NCBI GenBank Accession HA = MH559963 and NA = MH559965; BRMSA 1697). H1N1 and H1N2 viral samples were propagated in SPF (Specific Pathogen-Free) embryonated chicken eggs, and H3N2 virus was inoculated into Madin-Darby Canine Kidney (MDCK; BCRJ) cells, according to Zhang and Gauger [29]. To confirm the IAV presence, the cell supernatant and chorioallantoic fluid harvested from eggs were tested by hemagglutination assay [30] and by RT-qPCR [31].

### Virus concentration

Approximately 1 L of each virus was individually concentrated by tangential ultrafiltration using a flow pump coupled to a cassette containing a dialysis membrane consisting of polyethersulfone (PES) with a cut-off of 100 kDa (Vivaflow 200 System, Sartorius, Germany). Then, 50 mL of each viral concentrate was ultracentrifuged at 100,000 x g for 4 h at 4 °C (Optima LE 80 K, Beckman Coulter, USA). The supernatants were discharged, and the pellets of each virus were diluted to a final volume of 5 mL in TNE buffer (10 mM Tris, 100 mM NaCl and 1 mM EDTA, pH 7.4). The concentrated viruses were titrated by hemagglutination assay [30], and the hemagglutinin content was determined by SDS-PAGE (NuPAGE™ Bis-Tris, Thermo Fisher, USA) using acrylamide gel plate (4–12%) and silver staining according to the manufacturer’s recommendations. The HA concentration was calculated from the intensity of the bands (obtained with the ImageJ software) using an equation obtained from a standard calibration curve of albumin.

### Virosome preparation and characterization

The multivalent virosome was prepared according to de Jonge, Leenhouts [32] with modifications. Briefly, the same volume of each virus was mixed (1:1:1) and diluted in a 200 mM solution of 1,2-dicaproyl-sn-glycero-3-phosphocholine (DCPC, Avanti Polar Lipids, USA). After gentle homogenization, the pooled viruses were further diluted 1:2 (v/v) in the TNE buffer, and the final mixture was kept in an ice bath for 30 min to ensure the dissolution of the viral envelopes. Afterward, the mixture was ultracentrifuged at 100,000 x g for 30 min at 4 °C to remove the viral nucleocapsids. The supernatant was extensively dialyzed in a cellulose dialysis tube (cut-off 10 kDa, SpectraPor, USA) against TNE buffer for 48 h at 4 °C to remove the surfactant (DCPC), which led to the self-assembling of the virosome vesicles. Subsequently, the physical-chemical characteristics of the dialyzed virosome formulation were determined, measuring the particle size and zeta potential by dynamic laser scattering and laser Doppler electrophoresis (Zetasizer, Malvern). The HA antigen content from H1N1, H1N2, and H3N2 was determined by SDS-PAGE.

### Electron microscopy

Transmission electron microscopy (TEM) was used to examine the morphology and ultrastructure of the virosomes using the microscope JEOL JEM-1011 (Jeol, Japan). The samples were used pure or at a 50% dilution in ultrapure water. Before analysis, 3 µL of virosome suspension was deposited into a copper grid covered with Formvar®. The copper grids were fixed and after they had completely dried, they were contrasted with 1% Osmium Tetroxide vapor. Thus, 40 µL of Osmium Tetroxide were placed at the bottom of a petri dish containing the copper grids for 40 min. Virosomes were digitized using an UltraScan® camera connected to Digital Micrograph 3.6.5® computer software (Gatan, USA). To eliminate any doubt about what were virosomes and what were artifacts from the contrast with 1% Osmium Tetroxide, a negative control was created during the analysis that used only ultrapure water and Osmium Tetroxide [33].

### Assessment of the infectivity and cytotoxicity of influenza virosomes

In order to evaluate the infectivity of virosome, it was inoculated into embryonated chicken eggs and incubated at 37 °C for 4 days. Virosome was also inoculated into MDCK cells and monitored for 7 days.

For the in vitro cytotoxicity assays, immortalized macrophage lines (RAW 264.7 cells, BCRJ-0212) were cultured in complete Dulbecco’s Modified Eagle’s Medium (DMEM, Invitrogen) modified to contain 4 mM GlutaMAX (Gibco), 4500 mg/L glucose, 1 mM sodium pyruvate (Sigma-Aldrich), and 1500 mg/L sodium bicarbonate (Sigma-Aldrich) with 10% of fetal bovine serum (FBS) (Sigma-Aldrich). RAW 264.7 cells were cultured and maintained at 37 °C and 5% CO_2_. After the formation of the cell monolayer, the adherent cells were detached by scraping. Initially, RAW 264.7 cells (2 × 10^5^ cells/well) were seeded in 96-well plates, cultured for 24 h for adhesion and then treated with different dilutions of the virosome formulation (1:2, 1:4, 1:8, 1:16, 1:32, 1:64, 1:128 and 1:256, v/v) for 24, 48 and 72 h. This procedure was repeated for different passages of the cell culture until the desired sample size (*n* = 8) was reached. The control cells received the same volume of a simple liposome (prepared with phospholipids - Lipoid® S100, Lipoid). The cell viability was measured by the MTT (3-(4,5-dimethylthiazol-2-yl)-2,5-diphenyltetrazolium bromide, Sigma-Aldrich) assay. At the end of each incubation time, the medium was removed, and the cells were washed twice with DPBS (Sigma-Aldrich) and incubated for 3 h with 5 mg/mL MTT solution at 37 °C. After incubation, the precipitated formazan crystals were dissolved in 200 µL of dimethyl sulfoxide (DMSO, Sigma-Aldrich). Optical densities (OD) were measured at 540 nm using a Multiskan™ FC Microplate Photometer (Thermo Fisher Scientific). The absorbance values recorded for untreated cells (negative control) represent 100% of cell viability and were used as a reference to calculate the percentage of cell viability in the presence of each sample concentration. Complementary cytotoxicity analysis was performed using the enzyme terminal deoxynucleotidyl transferase (TdT) and the propidium iodide (PI) staining kit (APO-DIRECT™, BD Biosciences). RAW 264.7 cells were plated and treated with the virosome dilutions (1:32 and 1:64) as described above. The assay was done according to the manufacturer’s guidelines at 24, 48 and 72 h after exposure to the virosomes [33]. The staining protocol consisted of cell incubation with TdT-FITC enzyme and staining with propidium iodide. After 24 and 48 h of exposure, cells were analyzed using a flow cytometer (Accuri C6 Plus, Becton-Dickinson, USA), and the percentage of intact cell membranes per group was determined. The percentage of live cells was calculated from the fluorescence readings defined according to the kit instructions.

### Immunization of mice with multivalent influenza virosomes

The protocols and the use of animals for this research complied with the Animal Use Ethics Committee of Embrapa Swine and Poultry (protocol number 001/2016). C57BL/6 mice (female, 6–8 weeks old) were reared under SPF conditions and divided into 3 groups as follows: non-vaccinated control (NV, *n* = 20), intranasal vaccinated (5 µL/nostril or 10 µL/animal, IN, *n* = 20), intramuscular vaccinated (100 µL/animal, IM, *n* = 20). An additional group, non-vaccinated liposome control (*n* = 20), served as a control for virosomes. The G4 group did not show any difference in the analyzes performed when compared to the animals in the NV group (data not shown). A mucoadhesive adjuvant (carboxymethyl cellulose – CMC) was added to the formulation (0.125%, m/v) for intranasal administration, and Emulsigen-D® (MVP Adjuvants, USA) for intramuscular administration (20%, v/v) [34]. The experimental protocol consisted of the administration of two doses of the vaccine 2 weeks apart (days 1 and 15). At 21 days (day 36) and eight months later (day 255) after the second vaccine dose, ten animals/group from all three groups were euthanized using intraperitoneal injection of sodium pentobarbital (80 µg/g body weight).

### Biochemical determinations

For blood collection, mice were anesthetized with intraperitoneal ketamine-xylazin (ketamine 60 µg/g body weight, and xylazin 10 µg/g body weight). Blood samples were drawn by retro-orbital bleeding on days 0 (before vaccination), 3 and 17 (two days after each immunization). In order to assess the possible toxicity of the vaccine, quantification of biochemical markers from the serum samples was performed, evaluating the hepatic (AST and ALT) and renal (urea and creatinine) functions. These assays were performed with colorimetric kits, according to the manufacturer’s instructions (Labtest, Brazil).

### Morphologic assessment

For histopathology analysis, liver, kidney and lung tissue samples were collected at necropsy and fixed with 4% buffered paraformaldehyde, dehydrated in a graded series of ethanol, paraffin-embedded and sectioned at 4 μm. This material was stained with hematoxylin-eosin (H&E). Furthermore, to assess the in vivo cytotoxicity of the virosome, staining for apoptosis was performed using the In Situ Cell Death Detection kit (Roche, Germany), according to the manufacturer’s instructions. Nuclei were counterstained with 3,3-diaminobenzidine (Sigma-Aldrich, USA). The TUNEL assay is employed to identify and quantify apoptotic nuclei by an in situ reaction involving TdT-mediated dUTP-X nick end labeling. TUNEL-positive nuclei were quantified using light microscopy under magnification of 400x. The degree of TUNEL expression was calculated in 25 distinct fields (corresponding to a total area of 0.08 mm^2^). Results were expressed as cells/mm^2^.

### Bronchoalveolar lavage

Bronchoalveolar lavage fluid (BALF) from mice was obtained at necropsy (days 36 and 255), through a tracheostomy procedure in a biosafety cabinet. BALF was collected by flushing the lungs four times with 0.2 mL sterile physiological saline (0.9% NaCl) via the tracheal cannula. After BALF collection, a protease inhibitor cocktail was added to a final concentration of 1x and also phenylmethylsulfonyl fluoride (PMSF) to a final concentration of 1 mM. One portion of the BALF samples was stored at -80 °C for ELISA assay. ELISA was performed to quantify total IgA immunoglobulin in the BALF supernatant using the Invitrogen kit (Thermo Fisher, USA) in accordance with the manufacturer’s recommendations. Another part of the BALF samples was used for cell quantification. Trypan blue exclusion method using a Neubauer chamber was applied for cell quantification. For differential cytological analysis, a dried cell smear was prepared with an aliquot of the suspension, and stained with May-Grünwald-Giemsa staining. The slides were analyzed by light optical microscopy. At least 500 leukocytes were counted per high-power field, and the absolute differential cell counts were calculated by multiplying the percentage of each given cell type by the total cell count.

### Serology

Mice were anesthetized and blood was collected, through the retro-orbital plexus, at days 0, 36 and 255. Then, sera were evaluated for the presence of IAV-specific antibodies by hemagglutination inhibition (HI) assay [35]. The same IAV strains used in the virosomal vaccine composition were used as antigens in the HI assay. Results were reported as geometric mean antibody titers.

### Isolation of white blood cells from the spleen

The complete spleen from each mouse was aseptically collected in RPMI 1640 medium (Gibco) at necropsy (days 36 and 255). Each spleen was mechanically dissociated, and filtered through a nylon filter (70 μm). Then, red blood cells were lysed with Pharm Lyse™ buffer (BD Biosciences). The lysis reaction was stopped by adding of RPMI 1640 medium with 2% FBS, and the cells were washed twice. The cells were resuspended in complete RPMI 1640 medium, supplemented with 10% FBS (Gibco, Brazil), 1 mM GlutaMAX (Gibco, Brazil), 25 mM HEPES (Sigma-Aldrich, USA), 1 mM sodium pyruvate (Sigma-Aldrich, USA), 50 M 2-mercaptoethanol (Gibco, USA) and 100 U/mL penicillin-streptomycin (Sigma-Aldrich, USA). Finally, the cell number was counted with 0.4% trypan blue to determine the viable cell concentration. In general, the mice spleens yielded around 8–10 × 10^7^ viable splenocytes. The cells were resuspended in 95% FBS + 5% DMSO (Sigma-Aldrich) and cryopreserved at a final concentration of 2 × 10^6^ cells/mL.

### ***In vitro*** cell proliferation assay

Viable spleen cells were thawed and suspended in DPBS at a concentration of 5 × 10^6^ cells/mL and labeled with 2.5 µM carboxyfluorescein succinimidyl ester (CFSE) by applying the CellTrace™ CFSE Cell Proliferation kit (Invitrogen), according to previous reports [36]. After CFSE labeling, splenocytes were resuspended in complete RPMI 1640 medium, plated in 24-well plates (5 × 10^6^ cells/well). Subsequently, the cells were stimulated in vitro by adding 8000 TCID_50_/mL of the three vaccine viruses (H1N1, H1N2 and H3N2) for 96 h at 37 °C, under 5% CO_2_, in the dark. For the negative control, only culture medium was added to cells (non-virus-stimulated cells), and for the positive control, the cells were stimulated separately with 5 µg/mL of Concanavalin A from *Canavalia ensiformis* (Sigma-Aldrich). After in vitro stimulation of cells with H1N1, H1N2 and H3N2 viruses, lymphocyte proliferation from spleens was measured as an indicator of T and B-cell responses at 21 days after the boost immunization.

### Cell staining and flow cytometry

CFSE in combination with monoclonal antibodies (mAbs) enabled concomitant access to cell proliferation and activation status of cell subpopulations. Proliferation was detected by loss of CFSE fluorescence [36]. Flow cytometry analysis was performed to identify and quantify lymphocyte subpopulations (CD3e, CD4, CD8α, CD19, CD45R/B220 and sIgM mAbs), to measure the levels of cellular activation and mature resting marker expression (CD23, CD25 and CD69 mAbs), and cellular memory marker expression (CD62L and CD44 mAbs) (Becton Dickinson; Table 1). Cell densities were calculated and transferred to flow cytometry tubes (approximately 1 × 10^6^/well). Cells were treated with a blocking solution (10% v/v normal mouse serum) to block unoccupied binding sites on the second antibody, and thus cells were labeled for 30 min at room temperature in a dark room with a cocktail of specific mAbs (Table 1), 7-aminoactinomycin D (7-AAD) and isotype controls (BD Biosciences). Antibody concentrations used were in accordance with the manufacturer’s instructions.

**Table 1:** Panel of fluorochrome-labeled monoclonal antibodies

Panel	Antibody
Mouse Naïve/Memory T cell (cod 561,609, BD Becton Dickinson)	anti-CD44 / PE anti-CD4 / PerCP-Cy^TM^5.5 anti-CD62L / APC anti-CD3e / APC-CY^TM^7
Mouse T Lymphocyte Activation Antibody Cocktail (cod 557,908, BD Becton Dickinson)	anti-CD25 / PE-Cy™7 anti -CD69 / PE anti-CD3e / APC
Mouse T Lymphocyte Subset Antibody Cocktail (cod 558,431, BD Becton Dickinson)	anti-CD3e / PE-Cy™7 anti-CD4 / PE anti-CD8α / APC
Mouse B Lymphocyte Subset Antibody Cocktail (cod 558,332, BD Becton Dickinson)	anti-CD45R / B220 / PE-Cy™7 anti-CD23 (FcεRII) / PE anti-sIgM / APC
Mouse B Lymphocyte Activation Antibody Cocktail (cod 558,063, BD Becton Dickinson)	anti-CD25 / PE-Cy™7 anti-CD69 / PE anti-CD19 / APC

Monoclonal antibodies used to evaluate the cellular immune response of spleen cells from immunized mice in the in vitro stimulation assay

Before sample analysis, the flow cytometer settings were checked using Cytometer Setup and Tracking beads (CS&T beads, BD), as described in the manufacturer’s instructions. Compensation beads were used with single stains of each antibody to establish the compensation settings. The compensation matrix was identically applied to all samples. The side scatter (SSC) threshold level was set at 8,000 units to eliminate debris. Gates considered indicating positive and negative staining cells were set based on fluorescence minus one (FMO) tests of samples and these gates were applied consistently to each sample, allowing minor adjustments for SSC variability. 7-Aminoactinomycin D (7-AAD) staining was used to distinguish dead from viable cells by flow cytometry. In the preliminary procedure to set up instrument technical parameters, isotype controls were used to evaluate fluorochrome unspecific staining. Buffer for flow cytometry was prepared in PBS containing 0.01% w/v sodium azide (Sigma-Aldrich), 2% v/v FBS (Gibco) and 2% w/v bovine serum albumin (BSA, Sigma-Aldrich).

A total of 100,000 events per tube were acquired in the flow cytometer (Accuri C6 Plus and FACSCanto, Becton-Dickinson, USA) and analyzed using the FlowJo software (Becton-Dickinson, USA). The lymphocyte gate was set on light-scatter properties (Forward Scatter vs. Side Scatter). Proliferation by CFSE (reflected by successive reduction of fluorescence intensities by dye distribution to daughter cells) was measured by flow cytometry. Results were expressed as percentages of stained cells.

### Statistical analysis

Differences between vaccinated groups (intranasal – IN and intramuscular – IM routes) and non-vaccinated groups (NV) in biochemical data, immunoglobulins, apoptosis rate, and BALF cell count were analyzed through ANOVA, using the MIXED procedure of Statistical Analysis System (SAS - Cary, North Carolina, USA). In addition, differences between these groups in the in vitro cell proliferation assay were evaluated using the two-sided Student’s t test. Analysis of variance (F test) was carried out to assess the effect of the administration route and age in the in vitro cell proliferation assay, applying the Tukey test whenever a significant effect (*P* ≤ 0.05) of virosome was detected. For the analysis of HI, the descriptive level of probability of Fisher’s exact test was used; percentages followed by distinct letters on the lines differ significantly according to Fisher’s exact test. *P* values ≤ 0.05 were considered statistically significant [37].

## Results

### Multivalent virosome preparation, SDS-PAGE, and size distribution of particle diameters

The propagated influenza viruses were concentrated approximately 500x after concentration by ultracentrifugation and purification using ultrafiltration. Table 2 presents the hemagglutination titers and HA content of the IAVs prior to the preparation of the multivalent virosomes. A pre-formulation study was carried out, and we found that using equivalent volumes of each virus strain (1:1:1) led to a more stable formulation under refrigerated storage.

**Table 2:** Hemagglutination titer and HA content of IAVs used to produce the virosome vaccine formulation

Subtype	Strain identification	Hemagglutination titer	Hemagglutinin content (µg/mL) *
H1N1	A/swine/Brazil/25 − 15/2015	1:81920	36.2
H1N2	A/swine/Brazil/223-15-1/2015	1:40960	139.1
H3N2	A/swine/Brazil/28-15-8/2015	1:20480	478.9

* Hemagglutinin content was estimated by SDS-PAGE using a bovine serum albumin curve as standard for calculations

**Fig. 1:**
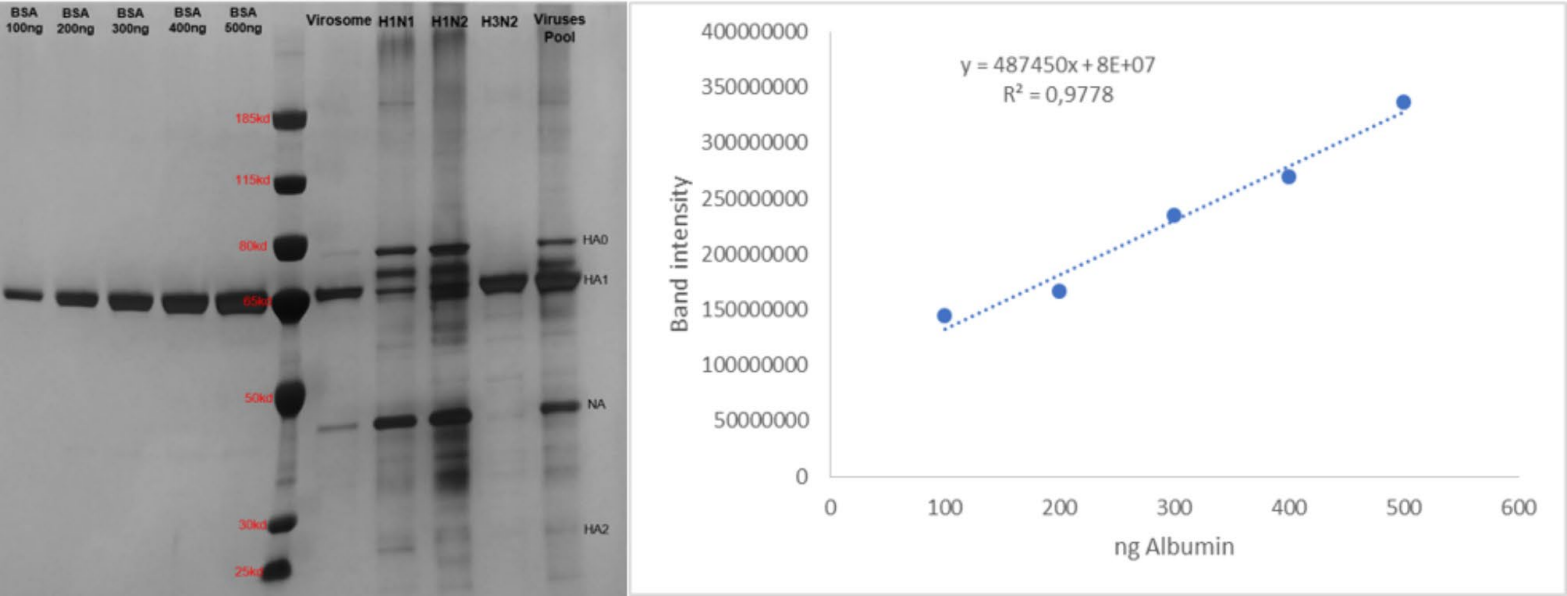
SDS-PAGE gel and standard curve of bovine serum albumin (approximately 66.5 kDa). Used to quantify the hemagglutinin of the viral strains and multivalent virosomes. On the SDS-PAGE gel it is visible: HA composed of two forms, uncleaved HA (HA0 ≈ 75–65 kDa) and cleaved HA (HA1 ≈ 55 kDa and HA2 ≈ 27 kDa); and NA (≈ 45–50 kDa). The band intensities were acquired using the ImageJ software

The purified virosomes were loaded on a polyacrylamide gel for electrophoresis. SDS-PAGE analysis (semi-quantitative method using albumin as standard; Fig. 1) showed that the purified virosomes contained mainly HA, based on the expected molecular weight of each IAV glycoprotein. The total HA content of the virosomes was nearly 160 µg/mL. Besides, considering the HA content for each virus, we estimated that the total HA presented in the formulation corresponds to 6, 21 and 73% for H1N1, H1N2 and H3N2, respectively, due to the differences on HA content for each strain.

**Fig. 2:**
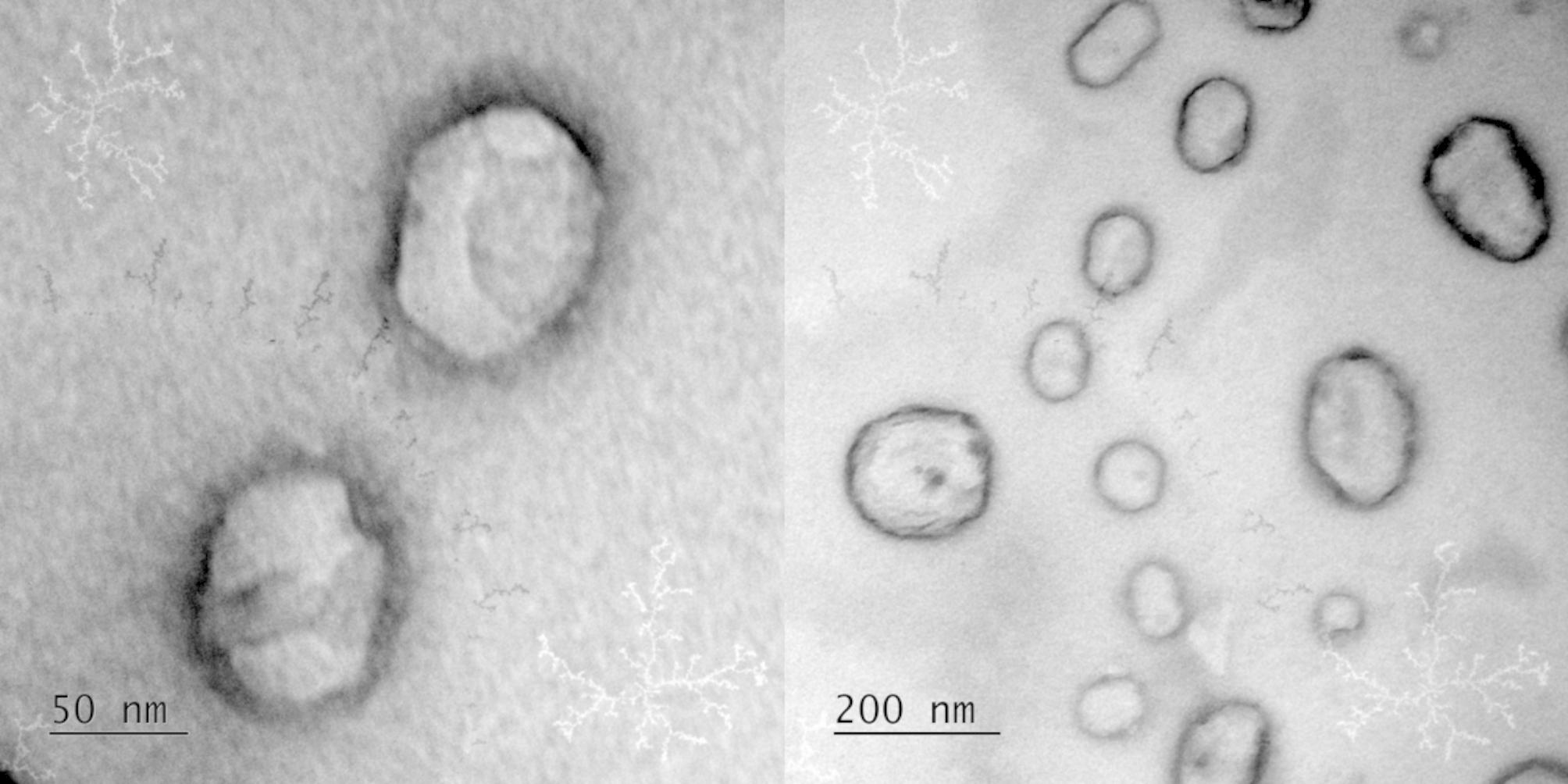
Transmission electron microscopy (TEM) image of multivalent virosomes produced with H1N1, H1N2 and H3N2 influenza strains. Image captured at 80 kV

The virosome formulation was characterized in terms of particle size and zeta potential, which were around 110 nm (PDI = 0.19) and − 6.2, respectively. TEM images displayed circular and ellipsoid structures as shown in Fig. 2. The virosomes presented an average diameter of 100 nm and unilamellar membrane, sometimes followed by blebs near it.

### In vitro cytotoxicity

At first, to ensure that the virosome consisted of a non-infective nanoparticle, the infectivity of virosome was assessed in embryonated chicken eggs and in MDCK cells. No viral replication was observed in embryonated chicken eggs inoculated with virosomes, and there was no visible cytopathic effect on cell line in comparison to control cells.

The viability of RAW 264.7 cells was evaluated after exposing the cells for 24, 48 and 72 h to different dilutions 1:2 to 1:256 (v/v) of the virosome (Table 3). After 24 and 48 h of incubation, the cells presented cell viability above 80% for all virosome dilutions, indicating the safety of virosomes. However, after 72 h of exposure of the cells to the virosome dilutions of 1:2 up to 1:8, cell viability ranged between 75 and 80%, showing that long-term exposure was slightly cytotoxic. Besides, when compared to the negative control in the MTT assay, exposure to the other virosome dilutions evaluated presented no effect on cell viability (> 80%) at this interval of incubation. In general, the virosome formulation was well tolerated at 1:16 to 1:256 dilutions at all exposure times. In terms of cell apoptosis, the cytotoxicity rate (apoptotic cells) determined by flow cytometry ranged from 1 to 2% for both virosome dilutions evaluated (1:32 and 1:64) during the same time periods (24, 48 and 72 h) [38].

**Table 3:** Cell viability of macrophages (RAW 264.7 cell line) exposed to different dilutions of the virosome formulation

Dilution of the virosome formulation	Time (hours)		
	24	48	72
1:256	98.33 ± 0.31 ^a^	95.97 ± 0.58 ^a^	91.11 ± 0.33 ^a^
1:128	97.51 ± 0.81 ^ab^	94.38 ± 0.88 ^a^	89.56 ± 0.68 ^ab^
1:64	97.75 ± 0.44 ^ab^	89.27 ± 0.64 ^b^	86.69 ± 0.61 ^b^
1:32	95.20 ± 0.96 ^abc^	90.23 ± 0.57 ^b^	87.48 ± 0.32 ^b^
1:16	94.15 ± 0.20 ^bc^	84.27 ± 1.05 ^c^	80.07 ± 1.09 ^c^
1:8	92.89 ± 0.21 ^c^	82.58 ± 0.43 ^c^	78.94 ± 0.22 ^cd^
1:4	93.25 ± 0.87 ^c^	82.48 ± 0.44 ^c^	77.95 ± 0.07 ^cd^
1:2	92.38 ± 0.88 ^c^	82.20 ± 0.63 ^c^	76.39 ± 0.75 ^d^
Pr > F	< 0.0001	< 0.0001	< 0.0001

Based on membrane damage (MTT assay) after 24, 48 and 72 h

Data represent mean ± standard error (*n* = 8). The analysis was performed with a one-way ANOVA and with Tukey’s post-test

^a,b,c,d^ Different superscript letters indicate significant statistical differences between virosome dilutions (*P* ≤ 0.0001)

### Biochemical and apoptosis analyses

No significant injury signs or differences were detected among non-vaccinated and vaccinated mice, regardless of the virosomal vaccine administration route (Table S1, Additional file 1). The hepatic (AST and ALT) and renal (urea and creatinine) values were within the normal range [39]. Furthermore, mice showed no adverse reactions associated with the immunization using the virosomes.

Regarding the histopathological analysis, the different tissues evaluated (kidney, lung and liver) did not show relevant microscopic changes. Furthermore, the TUNEL assay revealed no difference in the rate of cellular apoptosis in the kidney and liver of animals immunized with the virosome, which was similar to the control group at 36 days (Figure S1, Additional file 2).

### Cytological findings in BALF

On average, the BALF volume recovered was 82%. Mean values for total cell counts of BALF at 36 days from mice in the intranasal vaccinated, intramuscular vaccinated, and non-vaccinated groups were 8.8 ± 1.2 × 10^5^ cells/mL, 7.65 ± 0.9 × 10^5^ cells/mL, and 5.7 ± 0.7 × 10^5^ cells/mL, respectively. In all groups, the cellular composition of BALF consisted predominantly of alveolar macrophages (> 53%) and fewer numbers of lymphocytes (< 43%) and neutrophils (< 1%). Both groups of immunized mice (intranasally and intramuscularly) showed an increase in the population of lymphocytes (*P* ≤ 0.0001) when compared to the non-immunized animals (Table 4). Intranasally immunized mice also had a greater increase in total cell population.

**Table 4:** Differential leukocyte counts in the BALF of mice at 21 days after two doses of virosomes

Groups	Macrophages	Lymphocytes	Neutrophils
**Non-vaccinated**	5.06 ± 0.58 × 10^5^	0.30 ± 0.04 × 10^5b^	0.28 ± 0.14 × 10^5^
**Intranasally vaccinated**	4.74 ± 0.62 × 10^5^	3.70 ± 0.05 × 10^5a^	0.34 ± 0.10 × 10^5^
**Intramuscular vaccinated**	4.34 ± 0.94 × 10^5^	2.82 ± 0.04 × 10^5a^	0.42 ± 0.08 × 10^5^

BALF = bronchoalveolar lavage fluid

Values represent mean ± standard deviation (*n* = 10)

^a^ Different superscript letter indicates significant statistical differences between groups (*P* ≤ 0.0001). The analysis was performed with the Tukey test

### Immunogenicity assessment

**Fig. 3:**
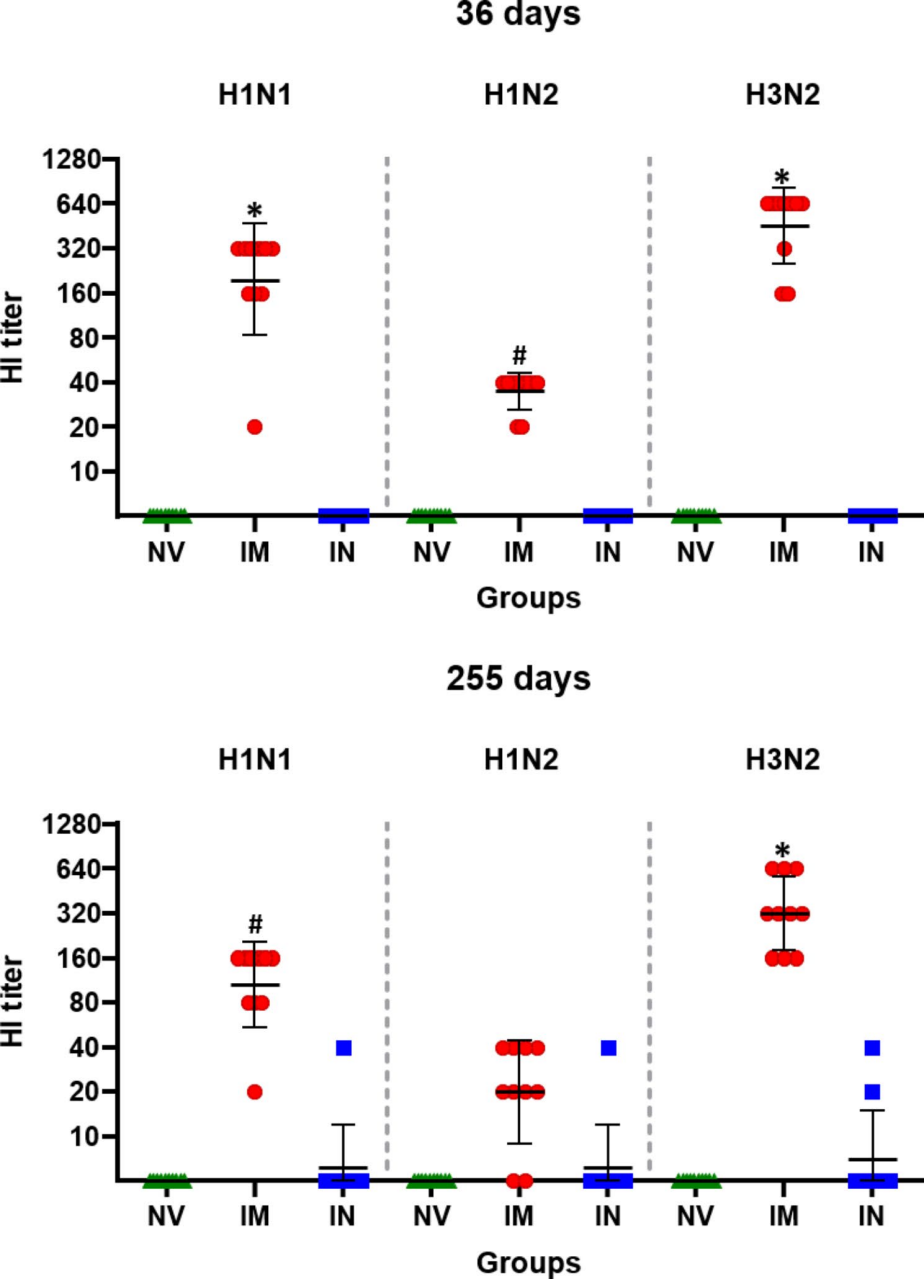
Hemagglutination inhibition (HI) assay. (NV) non-vaccinated, (IM) intramuscularly vaccinated, and (IN) intranasally vaccinated. Antibody titers by HI test for H1N1, H1N2 and H3N2 subtypes in serum samples from mice on day 21 and 240 post-vaccination. Data are shown for each mouse per group and the black lines represent the geometric mean titers ± standard deviation

Hemagglutination inhibition (HI) titers for the three IAV subtypes (H1N1, H1N2 and H3N2) were measured in serum samples obtained on days 21 (day 36) and 240 (day 255) after booster immunization for both intramuscular and intranasal immunization. The sera of mice immunized with virosomes by intramuscular route showed a significantly greater increase in HI titers than intranasal immunization for the three vaccine strains (*P* ≤ 0.0001; Fig. 3). In the follow-up evaluations (days 36), all mice in the intramuscularly vaccinated group had vaccine-induced HI antibody titers (˃1:40) to H3N2. The weak responses to H1N1 and H1N2 antibody titers (˂1:40) were detected in a few intramuscularly immunized mice. Nevertheless, the intramuscular vaccine was immunogenic in mice and elicited significant HI antibody responses to H1N1, H1N2 and H3N2. The seroconversion to intramuscular immunization 21 days after the booster immunization was 90% H1N1, 80% H1N2, 100% H3N2.

Intranasally vaccinated mice, on the other hand, did not develop detectable HI antibody titers 21 days after the booster immunization (day 36). Only one mouse vaccinated intranasally had HI-antibodies to H1N1 (titer 1:40), H1N2 (1:40) and H3N2 (1:40) viruses 8 months after the second immunization, as shown in Fig. 3. The intramuscular immunization elicited a greater rate of seroconversion (90% H1N1, 40% H1N2, 100% H3N2) compared with the intranasal immunization (10% H1N1, 10% H1N2, 10% H3N2) 8 months after the booster immunization.

**Fig. 4:**
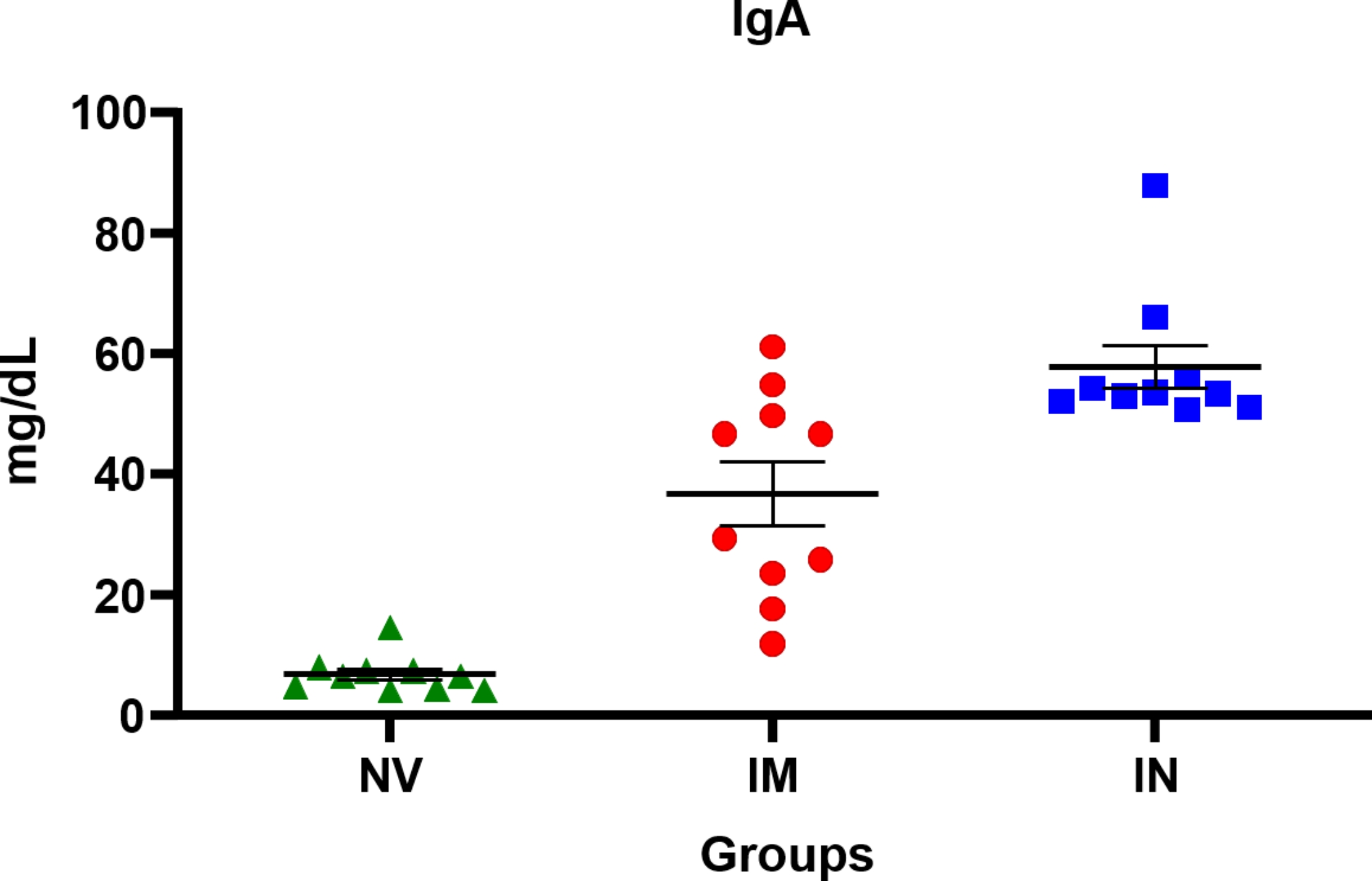
Quantification of total IgA antibody in the bronchoalveolar lavage fluid (BALF) of mice by ELISA assay. Data are shown for each mouse per group and the black lines represent the mean ± standard error. NV = non-vaccinated; IM = intramuscularly vaccinated; IN = intranasally vaccinated

The total immunoglobulin IgA was quantified in the BALF of mice. ELISA assays for total antibodies revealed that, regardless of the administration route, vaccinated mice had a higher IgA concentration than non-vaccinated mice (6.85 ± 0.98 mg/dL) on day 21 post-vaccination (*P* ≤ 0.0001). Animals intranasally vaccinated with the virosome (57.84 ± 3.61 mg/dL) had higher IgA concentrations than intramuscularly vaccinated mice (36.81 ± 5.39 mg/dL, *P* ≤ 0.0001; Fig. 4).

### Virosomes are an effective vaccine for inducing H1N1, H1N2 and H3N2-specific recall T-cell responses

To examine whether the virosome induces cell-mediated immunity, T cell suspensions were obtained from vaccinated mice on days 21 and 240 following the second vaccination. Gates were set using the non-virus-stimulated sample for each individual mouse. To summarize, the gate was based on forward scatter (FSC) and side scatter (SSC) features in order to estimate the lymphocyte population and exclude debris. The doublet cells were subjected to doublet plotting showing forward scatter height (FSC-H) against forward scatter area (FSC-A). Dead cells were excluded from the analysis using 7-AAD staining. The proliferation of lymphocytes was determined by CFSE^low^, which allowed the evaluation of the specific proliferation induced in each experimental group by each virus (H1N1, H1N2 and H3N2). Counterstaining with CD3e and CD45R/B220 allowed us to gate on T and B cells, respectively. Among T lymphocytes (CD3e^+^), subsets of CD4^+^ T cells, CD8α^+^ T cells, CD62L, CD44, CD25, and CD69 were distinguished using panels. In the first analysis, gates were used in the CD4^+^ and CD8^+^ populations for the analysis of T lymphocyte subgroups. In the second analysis, gates were used in the CD69^+^ and CD69^+^CD25^+^ populations, and in the third analysis, gates were used in the CD3e^+^CD4^+^CD44^high^CD62L^high^ and CD3e^+^CD4^+^CD44^high^CD62L^low^ populations. Among B-lymphocyte cells, activated lymphoblasts were stained with a mouse B-lymphocyte activation antibody cocktail and a mouse B-lymphocyte subset antibody cocktail.

The in vitro stimulated lymphocyte proliferation assay identified ten distinct cell subsets: CD3e^+^CD4^+^ (CD4^+^ T lymphocytes), CD3e^+^CD8α^+^ (CD8^+^ T lymphocytes), CD3e^+^CD69^+^ (very early activation T-cell), CD3e^+^CD69^+^CD25^+^ (effector T cells), CD3e^+^CD4^+^CD44^high^CD62L^high^ (central memory CD4^+^ T lymphocytes), CD3e^+^CD4^+^CD44^high^CD62L^low^ (effector memory CD4^+^ T lymphocytes), CD19^+^CD69^+^ (very early activation B-cell), CD19^+^CD69^+^CD25^+^ (effector B cells), CD45R/B220^+^sIgM^+^ (immature and mature B cells or transitional B cells), and CD45R/B220^+^sIgM^+^CD23^+^ (mature resting conventional B cells).

**Fig. 5:**
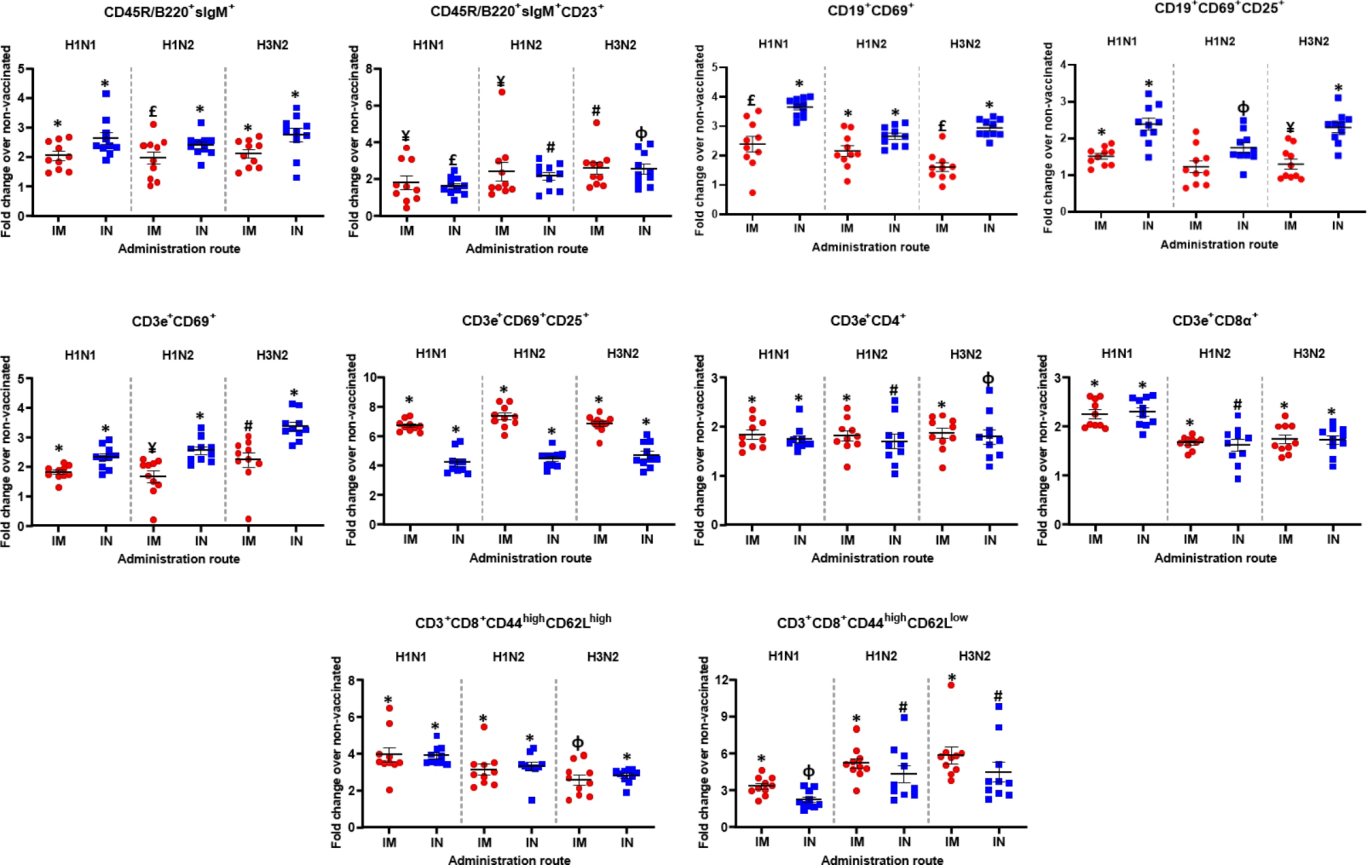
In vitro cell proliferation assay. Immune cells in the splenocytes proliferation assay stimulated with the vaccine viruses (H1N1, H1N2 and H3N2) were compared as a fold change from the intranasal (IN) and intramuscular (IM) vaccinated group over the non-vaccinated group (NV) on day 21 post-vaccination. Data shown are the fold increase in the mean percentage and standard error of indicated immune cells from vaccinated mice versus those of non-vaccinated (NV) mice. ^**¥**^*P* ≤ 0.05 versus NV, ^£^*P* ≤ 0.005 versus NV, ^#^*P* ≤ 0.001 versus NV, ^φ^*P* ≤ 0.0005 versus NV, and *P ≤ 0.0001 versus NV.

Notably, intramuscular and intranasal immunizations induced a robust T cell and B cell response (Fig. 5). For H1N1, H1N2 and H3N2 viruses, virosome-vaccinated (intramuscular and intranasal immunization) mice had more than 2-fold higher levels of T-cell subsets than non-vaccinated or naive mice (Fig. 5). Both intramuscular and intranasal immunization resulted in higher CD3e^+^CD4^+^, CD3e^+^CD8α^+^, CD3e^+^CD69^+^, CD3e^+^CD69^+^CD25^+^, CD3e^+^CD4^+^CD44^high^CD62L^high^, and CD3e^+^CD4^+^CD44^high^CD62L^low^ T cell subsets for H1N1 (*P* ≤ 0.0001), H1N2 (*P* ≤ 0.001) and H3N2 (*P* ≤ 0.001) (Fig. 5 and Table S2, Additional file 1). The administration route influenced the amount of some cell subsets, with intranasal immunization producing more CD3e^+^CD69^+^ cells (*P* ≤ 0.05) and intramuscular administration producing more CD3e^+^CD69^+^CD25^+^ cells (*P* ≤ 0.05; Table 5).

**Table 5:** H1N1, H1N2 and H3N2-specific recall T and B-cell responses after intramuscular and intranasal immunization with virosomes

Immune cells		36 days		255 days	
		Intramuscular	Intranasal	Intramuscular	Intranasal
		**H1N1**			
**B cells**	CD19^+^CD69^+^	2.394 ± 0.261	3.657 ± 0.098^a^	3.204 ± 0.134^b^	3.221 ± 0.182
	CD19^+^CD69^+^CD25^+^	1.515 ± 0.076	2.391 ± 0.162^a^	2.759 ± 0.102^b^	2.534 ± 0.200
	CD45R/B220^+^sIgM^+^	2.032 ± 0.149	2.615 ± 0.206^a^	2.399 ± 0.192	2.335 ± 0.201
	CD45R/B220^+^sIgM^+^CD23^+^	1.785 ± 0.350	1.601 ± 0.153	6.717 ± 0.530^b^	7.896 ± 0.554^b^
**T cells**	CD3e^+^CD4^+^	1.839 ± 0.091	1.743 ± 0.077	1.474 ± 0.053^b^	1.329 ± 0.070^b^
	CD3e^+^CD8α^+^	2.249 ± 0.088	2.293 ± 0.091	1.667 ± 0.082^b^	1.406 ± 0.137^b^
	CD3e^+^CD69^+^	1.809 ± 0.074	2.324 ± 0.119^a^	1.397 ± 0.082^b^	2.040 ± 0.070^a^
	CD3e^+^CD69^+^ CD25^+^	6.717 ± 0.122	4.210 ± 0.245^a^	1.882 ± 0.174^b^	1.299 ± 0.120^ab^
	CD3e^+^CD4^+^CD44^high^CD62L^high^	3.946 ± 0.393	3.893 ± 0.155	3.555 ± 0.205	2.526 ± 0.116^ab^
	CD3e^+^CD4^+^CD44^high^CD62L^low^	3.325 ± 0.237	2.231 ± 0.230^a^	1.938 ± 0.177^b^	1.226 ± 0.052^ab^
		**H1N2**			
**B cells**	CD19^+^CD69^+^	2.161 ± 0.184	2.663 ± 0.105^a^	2.749 ± 0.107^b^	2.037 ± 0.218^ab^
	CD19^+^CD69^+^CD25^+^	1.231 ± 0.161	1.748 ± 0.134^a^	2.141 ± 0.135^b^	1.875 ± 0.176^b^
	CD45R/B220^+^sIgM^+^	1.958 ± 0.219	2.397 ± 0.117^a^	2.641 ± 0.197^b^	2.156 ± 0.178
	CD45R/B220^+^sIgM^+^CD23^+^	2.393 ± 0.526	2.138 ± 0.221	12.70 ± 0.99^b^	12.51 ± 1.14^b^
**T cells**	CD3e^+^CD4^+^	1.813 ± 0.104	1.695 ± 0.148	1.332 ± 0.078^b^	1.313 ± 0.059^b^
	CD3e^+^CD8α^+^	1.664 ± 0.041	1.617 ± 0.120	1.074 ± 0.076^b^	1.035 ± 0.095^b^
	CD3e^+^CD69^+^	1.658 ± 0.201	2.543 ± 0.127^a^	1.504 ± 0.089	1.982 ± 0.092^a^
	CD3e^+^CD69^+^ CD25^+^	7.326 ± 0.234	4.458 ± 0.180^a^	1.347 ± 0.082^b^	1.097 ± 0.120^b^
	CD3e^+^CD4^+^CD44^high^CD62L^high^	3.136 ± 0.300	3.302 ± 0.236	3.518 ± 0.348	2.395 ± 0.130^ab^
	CD3e^+^CD4^+^CD44^high^CD62L^low^	5.184 ± 0.411	4.293 ± 0.683	2.893 ± 0.130^b^	2.087 ± 0.176^ab^
		**H3N2**			
**B cells**	CD19^+^CD69^+^	1.608 ± 0.148	2.942 ± 0.095^a^	2.703 ± 0.092^b^	2.421 ± 0.189^b^
	CD19^+^CD69^+^CD25^+^	1.303 ± 0.136	2.298 ± 0.136^a^	2.434 ± 0.132^b^	2.322 ± 0.126
	CD45R/B220^+^sIgM^+^	2.110 ± 0.142	2.735 ± 0.225^a^	2.712 ± 0.118^b^	2.426 ± 0.110
	CD45R/B220^+^sIgM^+^CD23^+^	2.558 ± 0.327	2.527 ± 0.282	10.63 ± 0.93^b^	13.02 ± 0.94^ab^
**T cells**	CD3e^+^CD4^+^	1.860 ± 0.107	1.793 ± 0.145	1.462 ± 0.054^b^	1.252 ± 0.068^b^
	CD3e^+^CD8α^+^	1.741 ± 0.093	1.724 ± 0.091	1.107 ± 0.054^b^	1.200 ± 0.085^b^
	CD3e^+^CD69^+^	2.226 ± 0.249^c^	3.361 ± 0.146^a^	2.088 ± 0.125	2.610 ± 0.122^ab^
	CD3e^+^CD69^+^ CD25^+^	6.848 ± 0.181	4.711 ± 0.264^a^	1.447 ± 0.176^b^	1.737 ± 0.254^b^
	CD3e^+^CD4^+^CD44^high^CD62L^high^	2.571 ± 0.268	2.796 ± 0.125	2.566 ± 0.178	2.138 ± 0.124^ab^
	CD3e^+^CD4^+^CD44^high^CD62L^low^	5.857 ± 0.679	4.471 ± 0.801	2.959 ± 0.120^b^	2.155 ± 0.110^ab^

Data are shown as fold change means ± standard errors

^a^ Superscript indicate a significant difference by the F test between the routes of administration within the age groups (*P* ≤ 0.05)

^b^ Superscript indicate a significant difference by the F test between ages groups within the routes of administration (*P* ≤ 0.05)

Mice immunized (intramuscular and intranasal) with virosomes showed a significant increase at 21 days’ post-vaccination (day 36) in the absolute numbers of effector (CD19^+^CD69^+^ and CD19^+^CD69^+^CD25^+^), transitional and mature B cells (CD45R/B220^+^sIgM^+^) compared to baseline (*P* ≤ 0.05) for H1N1, H1N2, and H3N2 (Fig. 5; Table 5). In contrast, intranasal immunization favored a greater establishment of B cells, CD45R/B220^+^sIgM^+^, CD19^+^CD69^+^ and CD19^+^CD69^+^CD25^+^ cell subsets, for H1N1, H1N2, and H3N2 than intramuscular immunization (Table 5).

### Virosomes induce efficiently long-lived immunity to influenza

Inducing long-lasting protective immunity is one of the objectives of vaccination. Hence, we assessed the longevity of H1N1, H1N2 and H3N2 antibody responses induced by virosome vaccination. The H1N1, H1N2 and H3N2-specific HI antibody responses to virosome-immunized mice were maintained at significantly higher levels (Fig. 5) than those of non-vaccinated mice for over 8 months, indicating that influenza virus immunity can be long-lived.

To assess the long-term protective efficacy, cellular immune responses were also analyzed in mice groups that were intramuscularly and intranasally immunized with multivalent virosomes at 8 months after vaccination.

As shown in Table 5 (comparison between routes of administration) and Table S2, Additional file 1 (comparison between non-vaccinated versus intramuscularly vaccinated group and non-vaccinated versus intranasally vaccinated group), 8 months after boost immunization, immunized mice showed a route-dependent increase of CD45R/B220^+^sIgM^+^CD23^+^ compared to the non-vaccinated group (*P* ≤ 0.0001). In comparison to non-vaccinated mice, intramuscular and intranasal immunized mice exhibited significant T- and B-cell proliferation in response to H1N1, H1N2, and H3N2 (*P* ≤ 0.05; Table S2, Additional file 1). However, mice immunized intranasally displayed a higher level of T-cell subset (CD3e^+^CD69^+^; Table 5) proliferation than mice immunized intramuscularly. The CD3e^+^CD4^+^CD44^high^ CD62L^high^ and CD3e^+^CD4^+^CD44^high^ CD62L^low^ cell subsets for H1N1, H1N2 and H3N2 were significantly higher when immunized intramuscularly than intranasally (*P* ≤ 0.05), as well as the CD3e^+^CD69^+^CD25^+^ and cell subsets for H1N1 (Table 5). Intramuscular immunizations induced greater memory-related central responses in tissues other than the respiratory mucosa, such as the spleen.

## Discussion

Designing novel vaccine candidates that closely mimic the native morphology of the specific virus without being pathogenic themselves remains a major challenge in the development of influenza vaccines [40]. Virosomes are tightly controlled virus-like particles that can be used in vaccine formulation [41]. In general, vaccination or infection fail conferring long-lasting protection due to the appearance of new or antigenically distinct influenza A virus strains [4]. Thus, the development of new, highly effective, and well-tolerated vaccines is essential. In a human safety and immunogenicity study, a prototype trivalent virosome influenza vaccine was compared to commercial whole inactivated virus and subunit vaccines [42]. The virosome vaccine produced higher protective titers and was less reactogenic and more immunogenic than either the whole-inactivated virus or subunit influenza vaccines. The synthesis of liposomes using envelopes from influenza A virus (virosome), which contain the main viral antigens (HA and NA), has yielded encouraging findings [4, 27]. In the current investigation, we designed a multivalent virosome from a cocktail of surface glycoproteins from H1N1, H1N2, and H3N2 viruses using the reconstitution technique with DCPC as detergent, and its immunogenicity as a swIAV vaccine candidate was investigated in mice. de Jonge, Holtrop [43] demonstrated that the use of DCPC as a viral membrane solubilizer has significant advantages over the conventional approach of virosome synthesis, which involves the solubilization of the viral envelope with Triton X-100, followed by its removal with polystyrene beads. Considering the high critical micelle concentration of DCPC, dialysis is an efficient procedure for the removal of DCPC, allowing the self-assembling of virosome vesicles with the benefit of not altering the viral antigens (HA and NA), a common issue related to Triton X-100 (alteration of the protein conformation and loss of antigenicity) [44, 45].

SDS-PAGE analysis revealed that multivalent virosomes contained both HA and NA protein bands and lacked virus nucleocapsid complexes. The average diameter of the virosomes was about 110 nm, which is consistent with the observations made in previous experiments [46]. Regarding the confirmation of HA (and to a lesser extent NA) by electrophoresis, this may result in the presentation of additional antigens by major histocompatibility complex classes I and II (MHC-I and MHC-II) proteins, resulting in the activation of T- and B-cells and dendritic cells, as seen during viral infection [47, 48]. In addition, influenza virosomes are a very promising strategy for antigen dose sparing, because it leads to high immunogenic response at low doses without affecting the protective effect of the vaccine [49]. We demonstrated that the swIAV virosomes are highly immunogenic and may elicit a strong humoral and cellular immune response against H1N1, H1N2 and H3N2 viruses.

Assessing the in vitro safety of virosome is essential to consider it as new vaccine prototype prior to its administration to animals [48]. For this, to assure that the virosome has no infectivity, the nanoformulation was inoculated in embryonated chicken eggs revealing the absence of viable virus, but with presence of HA agglutination activity suggesting that the virus disruption and virosome reconstitution have succeeded. In addition, cell viability assays were performed, revealing that no significant alterations were detected in any of the assessed virosome dilutions. This outcome served as confirmation of their safety for in vivo experiments.

The cytological examination of BALF revealed that the infiltration of lymphocyte cells into the airways was significantly higher in the vaccinated mice compared to the non-vaccinated mice. In previous studies, this lymphocyte infiltration in BALF suggested that the cellular immune response caused by the vaccination could be linked to the inflammatory cellular responses seen in the lungs [50]. However, the infiltration of lymphocytes into the bronchi and alveoli and lymphoid hyperplasia around the bronchi and blood vessels were not observed in the vaccinated groups as well as non-vaccinated group. In addition, no change in the rate of cellular apoptosis was observed in the lungs of vaccinated mice.

The literature lacks established operational definitions for the waning of influenza immunity; therefore, immunogenicity in this study was assessed using the criteria of the European Agency for the Evaluation of Medicinal Products (EMEA) [51]. Antibodies directed against the HA protein measured by hemagglutination inhibition (HI) assay are correlated with protection against influenza [3]. In the mouse model, even low levels of pre-existing influenza immunity (immunological memory) have an immunostimulating effect, as they enhance the antibody response against unrelated antigens delivered by influenza virosomes [48]. Hence, to confirm protective immunogenicity, at least one of the three EMEA requirements should be achieved: (i) seroconversion defined by ˃4-fold increase in HI antibody titer, reaching a HI titer of ≥ 1:40, in ˃40% of immunized subjects; (ii) an increase in geometric mean titers (GMT) of 2.5-fold; and (iii) seroprotection defined by the achievement of an HI titer of ≥ 1:40 in ˃70% of subjects. Liposomes containing no influenza virus proteins were administered to an additional control group (data not shown). This group exhibited similar HI antibody results as the non-vaccinated group. An important finding was that the polyvalent influenza-based virosome engendered protective HI titers against the three IAV strains that were significantly higher in the intramuscularly immunized mice compared to the intranasally immunized mice. According to EMEA criteria, seroconversion and seroprotection were achieved for three IAV strains by virosome administered via the intramuscular route. Regarding the fact that the mice used were specific pathogen-free and isogenic, even without the evaluation of antibodies prior to immunization, the fold-change was compared to the antibody titers of non-vaccinated mice, which were considered baseline levels. On the other hand, intranasally immunized mice had non-protective antibody titers at baseline, which were similar to previous studies [52]. Only one out of the intranasal-vaccinated animals displayed HI antibodies eight months after vaccination. According to this animal, to obtain a quality mucosal vaccine response against IAV, it will be necessary to reevaluate the formulation and consider new strategies for eliciting systemic and mucosal immune responses, including the use of appropriate vaccine adjuvants. Almost certainly, the antibody levels increased more gradually, the post-vaccination peak was later and was not detected in the initial analysis. However, our findings indicate that immunization with multivalent swIAV virosomes administered by nasal route is able to generate local immune responses, stimulating greater production of IgA in BALF, which may contribute to the protection of mice against a subsequent challenge. Therefore, intranasal immunization with the virosome-based swIAV did not induce a systemic humoral immune response but could induce a local humoral immune response. Besides, we highlight that the intranasal formulation consisted of a simple mucoadhesive system (inclusion of carboxymethyl cellulose), and possibly the immunological results could be improved with the design of a more sophisticated intranasal delivery system.

The lymphocyte proliferation from the spleen is significantly higher in the vaccinated group than in the non-vaccinated group. Our findings showed that the multivalent swIAV virosomal vaccine was able to induce effector, transitional, and mature B cells, as well as memory and effector T cells, with increased proliferation after virus stimulation. Nanoparticles and possibly virosomes often encapsulate ligand molecules for pattern recognition receptors (PRRs; e.g., Toll-like receptors, TLRs) that are expressed in dendritic cells and B cells [53, 54]. Expressed virosomes sometimes contain viral DNA or RNA, which has the potential to engage DNA or RNA sensors (i.e., TLR9 and TRL7) in B cells and dendritic cells [55]. Thus, we speculate that TLR signals might play antibody-enhancing roles during booster immunization of memory B cells in our experimental system. Further, nanovaccines may directly act on P-binding memory B cells; they eventually receive robust B cell receptor (BCR) signals or increased T cell help as a result of strong BCR cross-linking [54]. The stimulation of splenocytes from vaccinated mice with H1N1, H1N2 and H3N2 viruses also elicited higher cell proliferation of effector, transitional, and mature B cells under these conditions.

T-cell responses are known to help in the expansion of cross-protective immunity [56]. The cellular proliferation response of the liposome control group (data not shown) was the same as that of the non-vaccinated group. Our findings showed higher CD3e^+^CD4^+^ T helper, CD3e^+^CD8a^+^ T cytotoxic and CD3e^+^CD69^+^CD25^+^ T effector cells in vaccinated mice. The multivalent swIAV virosomes elicited a robust cytotoxic T lymphocyte (CTL) response mediated by CD8^+^ T lymphocytes, which is important for virus clearance [57]. The induction of a T-helper response is required for the antigen-specific B-cell and/or cytotoxic T-lymphocyte response to be supported [48]. Of note, intranasal and intramuscular immunization were associated with greater acquisition of CD69, residence marker associated with retention in lymphoid tissues [58].

Evidence observed in this study suggests that immunization with the virosome induced central memory and effector memory CD4^+^ T cells. These memory subsets, both short- and long-term, arise after antigenic stimulation with increased proliferative and reconstitutive capacities in immunized mice. Vaccine-induced memory T cells may be decisive in generating long-lasting immunity and inducing viral destruction. We observed that effector and central memory CD4^+^ T cells were abundant 21 days after booster immunization but declined over time, even though, they were still higher when compared to non-vaccinated mice. Thus, central memory CD4^+^ T cells induced by virosome immunization have a long duration and may be able to provide sustained help for CD8^+^ T cells [59]. The long-lived memory T cell population has an enhanced capacity for self-renewal and multipotency to generate all memory (central memory and effector memory) and effector T cell subsets in vitro [60]. Mice were vaccinated twice, which was sufficient to elicit an H1N1, H1N2, and H3N2 IAV-specific memory T-cell response, eliminating the need for heterologous prime-boost approaches. Therefore, avoiding continuous antigenic stimulation that can lead to progressive loss of memory potential, as an undesirable consequence, will drive T cells toward terminal differentiation, which compromises their capacity to clear systemic infections [61, 62]. The significant presence of memory cells after vaccination and their enhanced proliferative capacity can sustain the generation of all subsets of effector and memory T cells.

## Conclusions

These findings together have significant implications for the design of T cell–based vaccines that target intracellular pathogens, like influenza virus [60]. In addition, we suspect that the immunity measures observed here are lower than the described immunity demonstrated by viral challenge. HI is well accepted as a parameter to define protection induced by vaccination. Besides, HI quantifies the antibody response to the globular head of influenza hemagglutinin, but it does not evaluate the ability of the antibodies to neutralize virus infection [63, 64]. Nonetheless, there is a strong correlation between HI and functional viral neutralizing antibodies [63]. Finally, even if seroprotection is achieved in > 70% of isogenic mice vaccinated intramuscularly for subtypes H1N1, H1N2 and H3N2, future seroprotection studies in the target species (swine) are crucial. Antibody titers can fluctuate pre- and post-vaccination due to a variety of circumstances, including previous influenza infections and immunizations, genetic variations, and prior heterologous infections [63], but our mice had no antibody titers prior to the first immunization. According to the results obtained, the multivalent swIAV virosome designed was immunogenic in mice when intramuscularly administered, inducing systemic antibodies, and the potential for protection against influenza infection.

### Electronic supplementary material

Below is the link to the electronic supplementary material.

Supplementary Material 1

Supplementary Material 2

## Data Availability

The data that support this study are available from the corresponding author upon reasonable request. Source data are provided with this paper.

## Abbreviations

7-AAD: 7-aminoactinomycin D
BALF: Bronchoalveolar lavage fluid
BCR: B cell receptor
BSA: Bovine serum albumin
CFSE: Carboxyfluorescein succinimidyl ester
CMC: Carboxymethyl cellulose
CS&T: Cytometer Setup and Tracking
CTL: Cytotoxic T lymphocyte
DCPC: 1,2-dicaproyl-sn-glycero-3-phosphocholine
DMEM: Dulbecco’s Modified Eagle’s Medium
DMSO: Dimethyl sulfoxide
DPBS: Dulbecco’s phosphate-buffered saline
EMEA: European Medicines Evaluation Agency
FBS: Fetal bovine serum
FMO: Fluorescence minus one
FSC: Forward scatter
FSC-A: Forward scatter area
FSC-H: Forward scatter height
GMT: Geometric mean titers
HA: Hemagglutinin
H&E: Hematoxylin and eosin
HI: Hemagglutination inhibition
H1N1pdm: H1N1 pandemic
IAV: Influenza A virus
IM: Intramuscular
IN: Intranasal
mAbs: Monoclonal antibodies
MDCK: Madin-Darby canine kidney
MHC: Major histocompatibility complex
MTT: 3-(4,5-dimethylthiazol-2-yl)-2,5-diphenyltetrazolium bromide
NA: Neuraminidase
NV: Non-vaccinated
OD: Optical densities
PES: Polyethersulfone
PI: Propidium iodide
PMSF: Phenylmethylsulfonyl fluoride
PRR: Pattern recognition receptor
SAS: Statistical Analysis System
SPF: Specific pathogen-free
SSC: Side scatter
swIAV: Swine influenza A virus
TdT: Terminal deoxynucleotidyl transferase
TEM: Transmission electron microscopy
TLR: Toll-like receptor
TNE: Tris-NaCl-EDTA
VLP: Virus-like particle
WIV: Whole inactivated virus
