# Supplementary figures and images for: Immunological profile of mice immunized with a polyvalent virosome-based influenza vaccine
Source: Virol J. 2023 Aug 21;20:187. doi: 10.1186/s12985-023-02158-0 (PMC10463652; doi:10.1186/s12985-023-02158-0)

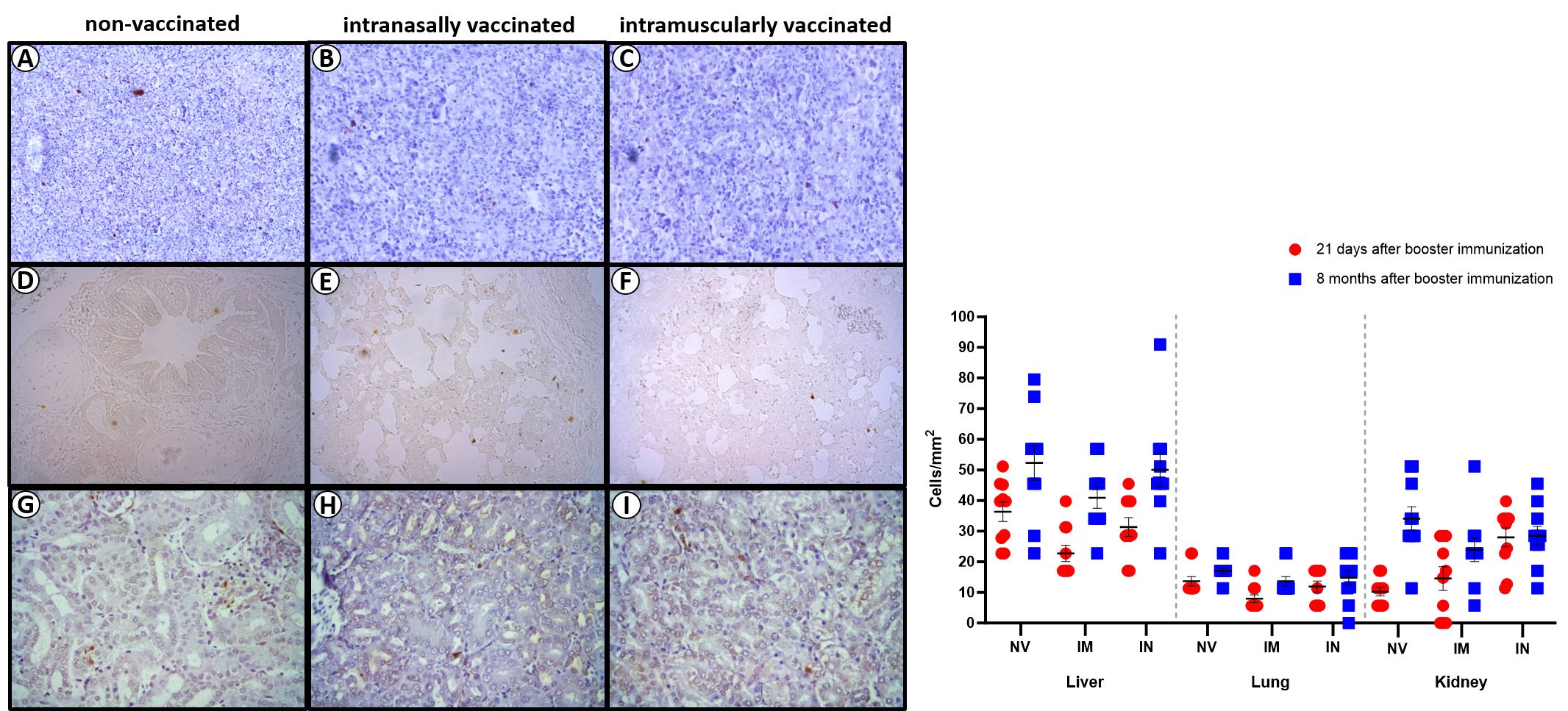

Supplement: Supplementary file 1 — Supplementary Material 1 [file 12985_2023_2158_MOESM1_ESM.png]
