# Supplementary material for: Immunological profile of mice immunized with a polyvalent virosome-based influenza vaccine
Source: Virol J. 2023 Aug 21;20:187. doi: 10.1186/s12985-023-02158-0 (PMC10463652; doi:10.1186/s12985-023-02158-0)
Supplement: Supplementary file 2 — Supplementary Material 2 [file 12985_2023_2158_MOESM2_ESM.docx]

**Table S1.** Biochemical parameters of renal and liver function of mice after two doses of multivalent vaccine for swine influenza.

| **Variable** |  | **Groups** |  | **Pr>F** |
| --- | --- | --- | --- | --- |
|  | **G1** | **G2** | **G3** |  |
| **ALT** | 50.76±2.05^b^ | 59.41±2.29 ^a^ | 56.31±2.22 ^ab^ | 0.0298 |
| **AST** | 80.30±5.06 | 71.35±4.78 | 73.90±5.08 | 0.4346 |
| **Creatinine** | 0.445±0.043 | 0.332±0.033 | 0.339±0.032 | 0.0669 |
| **BUN** | 17.19±1.29^b^ | 18.14±0.71^a^ | 18.70±0.7 ^a^ | <0.0001 |

Means followed by distinct letters in the lines differ significantly by Tukey's test (*P*≤0.05).

**Table S2.** Stimulation proliferation index for lymphocytes from splenocytes for H1N1, H1N2 and H3N2 among all groups at 21 days (52 days) and 8 months (255 days) after booster immunization.

| **Immune cells** |  | **Intramuscular** | **Intranasal** | | **Intramuscular** | | **Intranasal** | **Intramuscular** | | **Intranasal** |
| --- | --- | --- | --- | --- | --- | --- | --- | --- | --- | --- |
|  |  | **36 days** | | | | | | | | |
|  |  | **H1N1** | | **H1N2** | | | | **H3N2** | | |
| **B cells** | CD19^+^CD69^+^ | 2.394±0.261^d^ | 3.657±0.098^e^ | | 2.161±0.184^e^ | | 2.663±0.105^e^ | 1.608±0.148^b^ | | 2.942±0.095^e^ |
|  | CD19^+^CD69^+^CD25^+^ | 1.515±0.076^e^ | 2.391±0.162^e^ | | 1.231±0.161 | | 1.748±0.134^d^ | 1.303±0.136^a^ | | 2.298±0.136^e^ |
|  | CD45R/B220^+^sIgM^+^ | 2.032±0.149^d^ | 2.615±0.206^e^ | | 1.958±0.219^b^ | | 2.397±0.117^e^ | 2.110±0.142^e^ | | 2.735±0.225^e^ |
|  | CD45R/B220^+^sIgM^+^CD23^+^ | 1.785±0.350^a^ | 1.601±0.153^b^ | | 2.393±0.526^a^ | | 2.138±0.221^d^ | 2.558±0.327^c^ | | 2.527±0.282^d^ |
| **T cells** | CD3e^+^CD4^+^ | 1.839±0.091^e^ | 1.743±0.077^e^ | | 1.813±0.104^e^ | | 1.695±0.148^c^ | 1.860±0.107^e^ | | 1.793±0.145^d^ |
|  | CD3e^+^CD8α^+^ | 2.249±0.088^e^ | 2.293±0.091^e^ | | 1.664±0.041^e^ | | 1.617±0.120^c^ | 1.741±0.093^e^ | | 1.724±0.091^e^ |
|  | CD3e^+^CD69^+^ | 1.809±0.074^e^ | 2.324±0.119^e^ | | 1.658±0.201^a^ | | 2.543±0.127^e^ | 2.226±0.249^c^ | | 3.361±0.146^e^ |
|  | CD3e^+^CD69^+^ CD25^+^ | 6.717±0.122^e^ | 4.210±0.245^e^ | | 7.326±0.234^e^ | | 4.458±0.180^e^ | 6.848±0.181^e^ | | 4.711±0.264^e^ |
|  | CD3e^+^CD4^+^CD44^high^ CD62L^high^ | 3.946±0.393^e^ | 3.893±0.155^e^ | | 3.136±0.300^e^ | | 3.302±0.236^e^ | 2.571±0.268^d^ | | 2.796±0.125^e^ |
|  | CD3e^+^CD4^+^CD44^high^ CD62L^low^ | 3.325±0.237^e^ | 2.231±0.230^d^ | | 5.184±0.411^e^ | | 4.293±0.683^d^ | 5.857±0.679^e^ | | 4.471±0.801^e^ |
|  |  | **255 days** | | | | | | | | |
|  |  | **H1N1** | | | | **H1N2** | | | **H3N2** | |
| **B cells** | CD19^+^CD69^+^ | 3.204±0.134^e^ | 3.221±0.182^e^ | | 2.749±0.107^e^ | | 2.037±0.218^c^ | 2.703±0.092^e^ | | 2.421±0.189^e^ |
|  | CD19^+^CD69^+^CD25^+^ | 2.759±0.102^e^ | 2.534±0.200^e^ | | 2.141±0.135^e^ | | 1.875±0.176^c^ | 2.434±0.132^e^ | | 2.322±0.126^e^ |
|  | CD45R/B220^+^sIgM^+^ | 2.399±0.192^e^ | 2.335±0.201^e^ | | 2.641±0.197^e^ | | 2.156±0.178^e^ | 2.712±0.118^e^ | | 2.426±0.110^e^ |
|  | CD45R/B220^+^sIgM^+^CD23^+^ | 6.717±0.530^e^ | 7.896±0.554^e^ | | 12.70±0.99^e^ | | 12.51±1.14^e^ | 10.63±0.93^e^ | | 13.02±0.94^e^ |
| **T cells** | CD3e^+^CD4^+^ | 1.474±0.053^e^ | 1.329±0.070^c^ | | 1.332±0.078^b^ | | 1.313±0.059^d^ | 1.462±0.054^e^ | | 1.252±0.068^b^ |
|  | CD3e^+^CD8α^+^ | 1.667±0.082^e^ | 1.406±0.137^a^ | | 1.074±0.076 | | 1.035±0.095 | 1.107±0.054 | | 1.200±0.085^a^ |
|  | CD3e^+^CD69^+^ | 1.397±0.082^c^ | 2.040±0.070^e^ | | 1.504±0.089^d^ | | 1.982±0.092^e^ | 2.088±0.125^e^ | | 2.610±0.122^e^ |
|  | CD3e^+^CD69^+^ CD25^+^ | 1.882±0.174^c^ | 1.299±0.120^a^ | | 1.347±0.082^b^ | | 1.097±0.120 | 1.447±0.176^a^ | | 1.737±0.254^a^ |
|  | CD3e^+^CD4^+^CD44^high^ CD62L^high^ | 3.555±0.205^e^ | 2.526±0.116^e^ | | 3.518±0.348^e^ | | 2.395±0.130^e^ | 2.566±0.278^d^ | | 2.138±0.124^e^ |
|  | CD3e^+^CD4^+^CD44^high^ CD62L^low^ | 1.938±0.177^d^ | 1.226±0.052^b^ | | 2.893±0.330^d^ | | 2.087±0.176^d^ | 2.959±0.320^d^ | | 2.155±0.110^e^ |

Data are shown as fold change means ± standard error.

^a,b,c,d,e^ Different superscript letters indicate significant statistical differences between groups (a: *P*≤0.05, b: *P*≤0.005, c: *P*≤0.001, d: *P*≤0.0005, e: *P*≤0.0001).
